# Induced Release of a Plant-Defense Volatile ‘Deceptively’ Attracts Insect Vectors to Plants Infected with a Bacterial Pathogen

**DOI:** 10.1371/journal.ppat.1002610

**Published:** 2012-03-22

**Authors:** Rajinder S. Mann, Jared G. Ali, Sara L. Hermann, Siddharth Tiwari, Kirsten S. Pelz-Stelinski, Hans T. Alborn, Lukasz L. Stelinski

**Affiliations:** 1 University of Florida, Entomology and Nematology Department, Citrus Research and Education Center, Lake Alfred, Florida, United States of America; 2 Center for Medical, Agricultural, and Veterinary Entomology, Agricultural Research Service, U.S. Department of Agriculture, Gainesville, Florida, United States of America; University of Idaho, United States of America

## Abstract

Transmission of plant pathogens by insect vectors is a complex biological process involving interactions between the plant, insect, and pathogen. Pathogen-induced plant responses can include changes in volatile and nonvolatile secondary metabolites as well as major plant nutrients. Experiments were conducted to understand how a plant pathogenic bacterium, *Candidatus* Liberibacter asiaticus (Las), affects host preference behavior of its psyllid (*Diaphorina citri* Kuwayama) vector. *D. citri* were attracted to volatiles from pathogen-infected plants more than to those from non-infected counterparts. Las-infected plants were more attractive to *D. citri* adults than non-infected plants initially; however after feeding, psyllids subsequently dispersed to non-infected rather than infected plants as their preferred settling point. Experiments with Las-infected and non-infected plants under complete darkness yielded similar results to those recorded under light. The behavior of psyllids in response to infected versus non-infected plants was not influenced by whether or not they were carriers of the pathogen. Quantification of volatile release from non-infected and infected plants supported the hypothesis that odorants mediate psyllid preference. Significantly more methyl salicylate, yet less methyl anthranilate and D-limonene, was released by infected than non-infected plants. Methyl salicylate was attractive to psyllids, while methyl anthranilate did not affect their behavior. Feeding on citrus by *D. citri* adults also induced release of methyl salicylate, suggesting that it may be a cue revealing location of conspecifics on host plants. Infected plants were characterized by lower levels of nitrogen, phosphorus, sulfur, zinc, and iron, as well as, higher levels of potassium and boron than non-infected plants. Collectively, our results suggest that host selection behavior of *D. citri* may be modified by bacterial infection of plants, which alters release of specific headspace volatiles and plant nutritional contents. Furthermore, we show in a laboratory setting that this apparent pathogen-mediated manipulation of vector behavior may facilitate pathogen spread.

## Author Summary

In this investigation, we experimentally demonstrate specific mechanisms through which a bacterial plant pathogen induces plant responses that modify behavior of its insect vector. *Candidatus* Liberibacter asiaticus, a fastidious, phloem-limited bacterium responsible for causing huanglongbing disease of citrus, induced release of a specific volatile chemical, methyl salicylate, which increased attractiveness of infected plants to its insect vector, *Diaphorina citri*, and caused vectors to initially prefer infected plants. However, the insect vectors subsequently dispersed to non-infected plants as their preferred location of prolonged settling because of likely sub-optimal nutritional content of infected plants. The duration of initial feeding on infected plants was sufficiently long for the vectors to acquire the pathogen before they dispersed to non-infected plants, suggesting that the bacterial pathogen manipulates behavior of its insect vector to promote its own proliferation. The behavior of psyllids in response to infected versus non-infected plants was not influenced by whether or not they were carriers of the pathogen and was similar under both light and dark conditions. Feeding on citrus by *D. citri* adults also induced the release of methyl salicylate, suggesting that it may be a cue revealing location of conspecifics on host plants.

## Introduction

Transmission of plant pathogens by insect vectors is a complex biological process involving interactions between the plant, insect, and pathogen [1]–[2]. Pathogens can induce changes in the traits of their primary hosts as well as their vectors to affect the frequency and nature of interactions between hosts and vectors [3]–[13]. Plant morphology, as well as, primary and secondary plant compounds, including emitted volatiles and plant nutrients, are some of the traits that can be altered by pathogen infection of plants [14]–[16]. Fecundity, survival, and behavior are primary traits altered in insect vectors due to such infection [7]–[10], [12]–[13], [17]–[21]. Plant pathogen infection may alter both plant morphology and chemistry; therefore, research efforts have focused on the vector's response to such changes in their plant host [7]–[12], [18]–[21].

*Candidatus* Liberibacter asiaticus (Las) is a gram-negative, fastidious, phloem-limited bacterium that causes huanglongbing (HLB) disease in citrus [20]. Based on their 16S rRNA gene sequence, three species of the pathogen have been identified to date: 1) *Candidatus* Liberibacter asiaticus (Las), 2) *Candidatus* Liberibacter africanus (Laf), and 3) *Candidatus* Liberibacter americanus (Lam) [22]–[26]. Each species can be graft-inoculated into plants with infected plant tissue [22]–[23], [27]–[29]. Las and Lam are transmitted by the Asian citrus psyllid, *Diaphorina citri* Kuwayama (Hemiptera: Psyllidae); whereas, Laf is transmitted by the African citrus psyllid, *Trioza erytreae* (Del Guercio) (Hemiptera: Psyllidae). Both *D. citri* adults and nymphs are capable of transmitting Las after feeding on an infected plant for 30 minutes or longer [19], [30]–[32]. If the pathogen is acquired by psyllid nymphs, adults are capable of transmitting the bacteria immediately after emergence [19], [30], [33]. The bacteria presumably multiply within the vector and are retained throughout the entire life cycle of the vector [33]–[34].

HLB, otherwise known as citrus greening disease, is one of the most devastating diseases of citrus worldwide [22], [28], [35]. HLB affects plant phloem, causing yellow shoots, mottling, chlorosis, and twig die back that result in rapid tree decline and ultimately tree death. In areas of the world where HLB is endemic, citrus trees decline and die within a few years and may never produce usable fruit [28]. All commercial citrus cultivars are susceptible to HLB and there is no cure currently. HLB spread is dependent upon the density and movement of *D. citri* from infected trees to uninfected trees [36]. Mating and oviposition in *D. citri* occurs exclusively on the new and tender flush of its host plants [37]–[38]. In addition, *D. citri* is known to use combined olfactory and visual cues to locate citrus host plants [39]–[40].

Many plant species emit volatiles in response to arthropod feeding or pathogen attack [41]–[43]. Plants can defend themselves by producing infochemicals or toxins that act directly against herbivores or indirectly by producing chemicals, which attract herbivore natural enemies [44]–[49]. Several metabolic pathways are activated in plants when pathogens invade [49]–[50]. Some metabolites accumulate in leaves, whereas others are only produced in a particular type of induction [50]–[53]. For example, biosynthesis of the phenolic defense hormone, salicylic acid (SA), and its conversion to derivatives such as salicylic acid β-glucoside, salicylic acid glucose ester, and methyl salicylate (MeSA), are major and general metabolic events in pathogen-infected plants [50], [54]–[55]. Such plant hormone-mediated responses affect the interactions between pathogens and herbivores on plants in many ways [56].

Pathogens can also induce the release of chemicals from plants that benefit their survival and spread or suppress plants' defense responses [16], [57]. For example, infection of apple with *Candidatus* Phytoplasma mali, (causal agent of apple proliferation) induces release of β-caryophyllene, which attracts apple psyllids (*Cacopsylla picta*) to infected trees and facilitates pathogen spread [16]. Similarly, cucumber mosaic virus (CMV)-, potato leaf roll virus (PLRV)- and barley yellow dwarf virus (BYDV)-infected plants are more attractive to their aphid vectors, *Aphis gossypii, Myzus persicae* and *Rhopalosiphum padi* or *Schizaphis graminum*, than non-infected plants due to induced production of volatiles as a result of infection [3]–[4], [58]–[59]. CMV was reported to reduce the quality of squash plants for *M. persicae* and *A. gossypii* [9]. Similarly, BYDV reduced the quality of wheat plants for *R. padi* [12]. In contrast, PLRV improved the quality of potato plants as hosts to its vector, *M. persicae* [13].

Although most investigations of plant-pathogen interactions have focused on individual aspects of pathogen-induced plant defense [50], [54]–[55] or the response of insect vectors to infected versus non-infected plants [9], [15]–[16], studies are emerging which highlight multiple interactions between herbivores and phytopathogens [9], [47], [56], [60]. The goal of current research was to understand the interactions between the pathogen (Las), the vector (*D. citri*), and the host plant (citrus) to determine whether pathogen-induced plant response may facilitate spread of the HLB pathogen. The specific objectives were to determine whether pathogen infection alters the attractiveness of host plants to the vector and to determine how infection of the vector with the pathogen affects its behavioral response. To assess the effects of Las infection on vector-plant interactions, we conducted olfactometer and gas chromatography-mass spectroscopy (GC-MS) assays to identify specific olfactory cues attractive to psyllid vectors. In addition, we evaluated the effect of olfactory cues on the settling and dispersal behaviors of psyllids. Our results indicate apparent pathogen-mediated manipulation of vector behavior, which may promote pathogen spread.

## Results

### Response of psyllids to host plant odors

**Figure 1:**
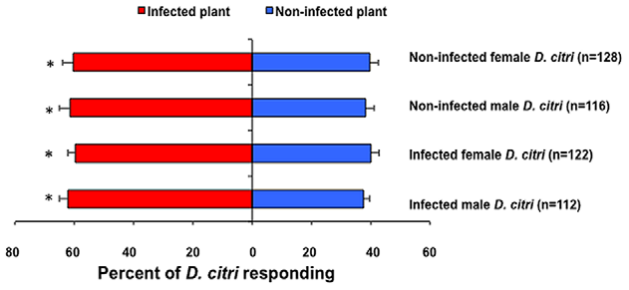
Response of *D. citri* to odors emitted from Las-infected versus non-infected citrus in a laboratory olfactometer. Bars labeled by an asterisk are significantly different (χ^2^ test, p<0.05). n = total number of psyllids that responded.

The majority (>80%) of those *D. citri* tested responded to the odors of either non-infected or Las-infected citrus plants. Significantly more *D. citri* males (χ^2^ = 4.32, df = 1, P = 0.04) and females (χ^2^ = 6.53, df = 1, P = 0.01) were attracted to the odors from Las-infected plants than non-infected plants (Fig. 1). Las-infected male (χ^2^ = 5.24, df = 1, P = 0.02) and female (χ^2^ = 4.37, df = 1, P = 0.04) psyllids were also more attracted to the odors from infected than non-infected plants (Fig. 1). No significant differences were detected when *D. citri* adults were exposed to non-infected grafted (sham grafted) vs. non-grafted-non-infected plants (χ^2^ = 1.51, df = 1, P = 0.22).

### Host plant selection under light or dark conditions

#### Light conditions

**Figure 2:**
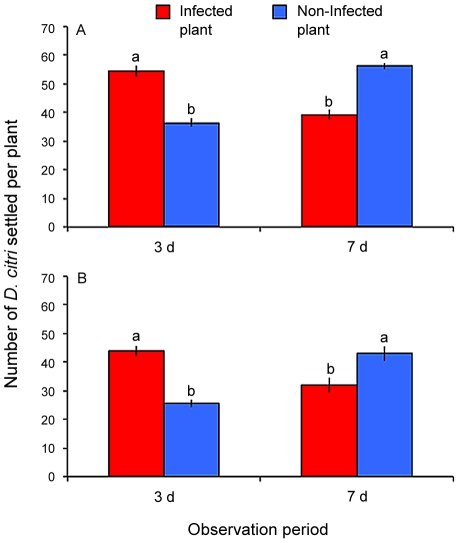
Settling preference of combined non-infected and Las-infected *D. citri* on Las-infected versus non-infected citrus plants. Panel (A) shows response under light conditions and panel (B) shows response under dark conditions. Bars with the same letter are not significantly different (Tukey's HSD test, p<0.05).

Observation time and Las infection status of *D. citri*, and gender of *D. citri* did not significantly affect the number of *D. citri* that settled on Las-infected versus non-infected plants (F = 2.63, df = 1,30, P = 0.12 and F = 0.09, df = 1,24, P = 0.76, respectively). However, significantly more female *D. citri* landed on Las-infected than on non-infected plants (F = 5.70, df = 1,24, P = 0.03). Las infection status of plants significantly interacted with observation time (F = 160.07, df = 1,30, P<0.001). More adult *D. citri* (of either gender and infection status) landed upon Las-infected plants than on non-infected plants three d after release (Fig. 2A). However, more adult *D. citri* were found on non-infected plants than on Las-infected plants seven d after release (Fig. 2A). Non-infected plants remained Las-negative when examined four months after exposure to Las-infected or non-infected *D. citri* in behavioral assays.

#### Dark conditions

Observation time, Las infection status of *D. citri*, and gender of *D. citri* did not significantly affect the number of *D. citri* that settled on Las-infected versus non-infected plants (F = 1.67, df = 1,30, P = 0.21; F = 3.36, df = 1,24, P = 0.08; and F = 1.70, df = 1,24, P = 0.21, respectively). Las infection status of plants significantly interacted with observation time (F  = 49.45, df = 1,30, P<0.001). More adult *D. citri* (of either gender or infection status) landed on Las-infected plants than on non-infected plants three d after release (Fig. 2B). However, more adult *D. citri* were found on non-infected plants than on Las-infected plants seven d after release (Fig. 2B). Non-infected plants remained Las-negative when examined four months after exposure to Las-infected or non-infected *D. citri* in behavioral assays.

### Movement of *D. citri* between Las-infected and uninfected plants

**Figure 3:**
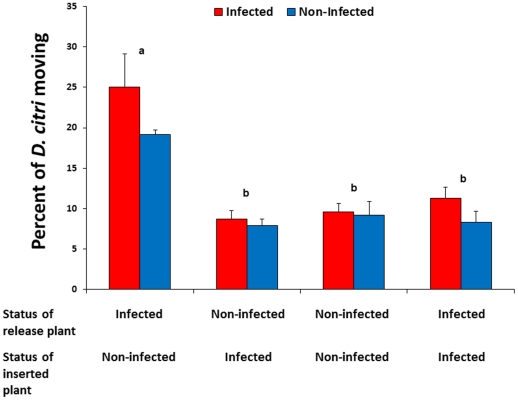
Movement of previously settled *D. citri* from Las-infected to non-infected citrus plants. Red bars show the response of Las-infected psyllids and blue bars show the response of non-infected psyllids; no significant differences were detected between psyllid types within plant treatments. Pairs of bars representing plant treatment combinations labeled with different letters are significantly different from one another (Tukey's HSD test, p<0.05).

Movement of non-infected or Las-infected *D. citri* from Las-infected to non-infected plants was greater than observed for any other treatment combination (F = 24.95, df = 3,24, P<0.0001) (Fig. 3). More Las-infected *D. citri* tended to move than uninfected counterparts after initial settling, although this difference was not statistically significant (F = 3.70, df = 1,24, P = 0.07). Las infection status of psyllids did not interact significantly with the direction of movement between plant treatments (F = 0.91, df = 3,24, P = 0.45). Approximately 5% of non-infected psyllids moving from infected to non-infected plants acquired the Las bacterium (Table 1). Four months after the termination of the experiment, all of the non-infected plants that had been inserted into cages with Las-positive plants tested positive for Las (Table 1).

**Table 1:** Las infection status of inserted plants and *Diaphorina citri* migrating from initial point of forced settling to subsequently inserted plant treatments.

Initial plant	Inserted plant	Las infection status of released *D. citri*	% of inserted plants infected with Las	% of Las-infected *D. citri* on inserted plants
Non-infected	Infected	Non-infected	100	0.0
Non-infected	Infected	Infected	100	71.2
Non-infected	Non-infected	Non-infected	0.0	0.0
Non-infected	Non-infected	Infected	100	68.9
Infected	Infected	Non-infected	100	6.6
Infected	Infected	Infected	100	76.3
Infected	Non-infected	Non-infected	0.0	4.8
Infected	Non-infected	Infected	100	69.7

### Nutritional status of Las-infected and non-infected citrus plants

There were marked differences between infected and non-infected plants with respect to nutrient content (Table 2). Las-infected plants were deficient in nitrogen, phosphorus, magnesium, zinc, and iron as compared with non-infected plants (Table 2). However, Las-infected plants had higher potassium and boron contents compared with non-infected plants (Table 2). There were no differences between Las-infected and non-infected plants for calcium, sulfur, silicon, manganese, sodium, molybdenum, aluminum, and chlorine.

**Table 2:** Differing levels of various nutrients between Las-infected and non-infected *Citrus sinensis* plants.

Plant status	N	P	K	Mg	Ca	S	B	Zn	Mn	Fe	Si	Na	Mo	Al
Uninfected plant	3.13	0.17	3.00	0.43	1.88	0.25	46.67	29.33	39.00	86.33	0.15	0.08	0.02	35.34
Las-infected plant	2.32	0.11	4.05	0.29	1.75	0.24	76.00	19.67	35.33	66.67	0.16	0.11	0.01	16.98
t value	8.03	3.72	19.10	8.67	1.67	0.42	12.35	45.85	1.21	16.75	0.43	1.69	0.42	2.08
P value	<0.01*	0.01	<0.01*	<0.01*	0.15	0.69	<0.01*	<0.01*	0.28	<0.01*	0.68	0.14	0.69	0.09

Nutrient values in columns labeled with * are significantly different at p<0.05 (two-sample t-test). Values for nitrogen (N), phosphorus (P), potassium (K), manganese (Mg), Calcium (Ca), Sulfur (S), silicon (Si), and sodium (Na) are in % while values for boron (B), zinc (Zn), manganese (Mn), iron (Fe), molybdenum (Mo), and aluminum (Al) are in ppm.

### Feeding efficiency of *D. citri* on non-infected versus infected plants

**Figure 4:**
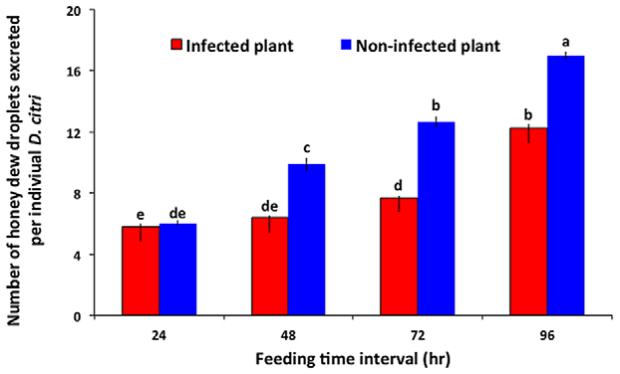
Feeding efficiency of *D. citri* on Las-infected versus non-infected citrus leaves as measured by honeydew excretion. Bars labeled with different letters are significantly different from one another (Tukey's HSD test, p<0.05).

The number of honey dew droplets produced by psyllid feeding, a surrogate measure of feeding efficiency, was significantly affected by the infection status of plants (F = 65.99, df = 1,39, P<0.0001), feeding exposure time (F = 81.81, df = 3,24, P<0.0001), and the interaction between the two factors (F = 7.11, df = 3,28, P = 0.0011). There was no significant difference in the amount of feeding on non-infected versus infected plants after 24 h of feeding by psyllids (Fig. 4). However, psyllids fed significantly more on non-infected than on Las-infected plants after 48, 72 and 96 h, respectively.

### Volatile release by Las-infected and non-infected citrus plants

**Figure 5:**
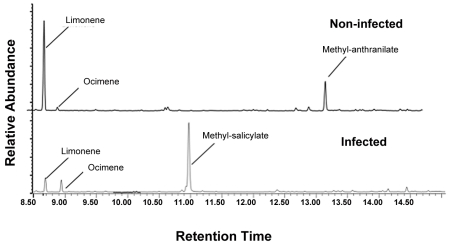
Chromatograms displaying volatile differences between Las-infected and non-infected plants. Release of methyl salicylate was significantly greater from plants infected with Las, while release of D-limonene and methyl anthranilate was significantly greater from non-infected plants.

There were significant qualitative and quantitative (Table 3, Fig. 5) differences between the headspace volatiles of non-infected and Las-infected plants (Table 3). Las-infected plants released significantly more MeSA than non-infected plants (Table 3, Fig. 5), while non-infected plants released more methyl anthranilate (MeAN) and D-limonene than infected plants (Table 3). Using a Random Forests algorithm, a minimum of two compounds, MeAN and MeSA, were identified that discriminated between the infected and non-infected volatile organic compound (VOC) signatures of these treatments, with an estimate prediction error of 0.15 and a ‘leave-one-out’ bootstrap error of 0.20.

**Table 3:** Volatiles from Las-infected and non-infected citrus plants presented as average percentage ±1 standard error (SE) of n-octane equivalents of volatile organic compounds collected from plants' headspace.

Compound	RT	CAS#*	Uninfected (Mean % ± SE)	Las-infected (Mean % ± SE)	t-value (df)	P-value
Sabiene	7.94	3387-41-5	11.0±2.54	15.0±0.67	−0.55 (8)	0.60
β-pinene	8.01	127-91-3	0.01±0.001	0.01±0.001	−0.135 (8)	0.90
Myrcene	8.2	123-35-3	3.0±0.44	3.0±0.23	0.27 (8)	0.79
3-Carene	8.57	13466-78-9	5.0±1.09	2.0±0.08	1.12 (8)	0.39
**D-limonene**	**8.71**	**5989-27-5**	**59.0±3.18**	**27.0±0.79**	**3.67 (8)**	**0.01** *
β-ocimene	8.95	502-99-8	6.0±1.01	6.0±0.5	−0.30 (8)	0.77
Linalool	9.83	78-70-6	2.0±0.56	1.0±0.11	0.48 (8)	0.65
Menthatriene (1,3,8-para)	10.14	18368-95-1	0.01±0.001	4.0±0.25	−2.21 (8)	0.06
**Methyl salicylate**	**11.01**	**119-36-8**	**1.0±0.21**	**39.0±2.86**	**−3.06 (8)**	**0.02** *
Geranial	12.21	141-27-5	2.0±0.45	0.0±0.0	2.10 (8)	0.07
**Methyl anthranilate**	**13.9**	**134-20-3**	**12.0±1.25**	**0.01±0.05**	**3.24 (8)**	**0.01** *
Caryophyllene	14.45	87-44-5	0.01±0.02	3.0±0.22	−2.06 (8)	0.07

Each compound is characterized by its retention time (RT) and major ion.

Bold values indicate a significant difference between treatments (*P*<0.05). The chemicals which were present in different proportions between Las-infected and non-infected citrus plants are shown. Identification was based on comparisons of retention times with standard and spectral data from Adams, EPA, Nist05 Libraries and synthetic standard comparison.

*: CAS #: Chemical Abstract Service number.

### Volatile release before and during psyllid feeding

A significant amount of MeSA was detected in headspace volatiles from plants after psyllids were introduced and allowed to feed, as compared with headspace volatiles from plants prior to psyllid feeding (t = −2.97, df = 5, P = 0.03). MeSA was detected in headspace profiles from plants infested with female as well as male psyllids; however, there was no significant difference in the amount of MeSA released in response to male verses female feeding (t = 0.27, df = 4, P = 0.80). Analysis of the volatile profiles of plants prior to the introduction of psyllids of either sex or from negative control plants yielded no MeSA. The amount of MeSA released (mean ± SE) by plants exposed to psyllid feeding (10.3±3.00 ng/plant/24 hr) was similar to the amount released by Las-infected plants (12.87±4.78 ng/plant/24 hr), and both of these amounts were similar to the quantity of synthetic MeSA found to be attractive to psyllids in behavioral bioassays (Table 4).

**Table 4:** Responses of *Diaphorina citri* when assayed with synthetic volatiles identified from Las-infected and non-infected citrus plants.

Chemical	Dosage (µg)	Proportion of *D. citri* responding to treatment arm	Proportion of *D. citri* responding to control arm	χ^2^ value	P value
†β-ocimene (positive control)	0.001	52.22	47.78	0.18	0.67
	0.01	54.46	45.54	0.8	0.37
	0.1	53.19	46.81	0.38	0.54
	1	60.44	39.56	3.97	0.05*
	10	61.46	38.54	5.04	0.02*
	100	62.77	37.23	6.13	0.01*
D-limonene	0.001	56.06	43.94	0.97	0.32
	0.01	60.87	39.13	2.17	0.14
	0.1	54.39	45.61	0.44	0.51
	1	54.65	45.35	0.74	0.39
	10	60.24	39.76	3.48	0.06
	100	61.54	38.46	4.85	0.03*
Methyl salicylate	0.001	54	46	0.32	0.57
	0.01	60.44	39.56	3.97	0.04*
	0.1	54.76	45.24	0.38	0.54
	1	45.12	54.88	0.78	0.38
	10	41.03	58.97	2.51	0.11
	100	39.56	60.44	3.97	0.04*
Methyl anthranilate	0.001	58.06	41.94	0.81	0.37
	0.01	57.14	42.86	0.71	0.4
	0.1	61.76	38.24	1.88	0.17
	1	57.33	42.67	1.61	0.2
	10	56.1	43.9	1.22	0.27
	100	52.27	47.73	0.23	0.63

**†:** β-ocimene carried 20–25% limonene. P values labeled with* are significantly different (χ^2^ test, *P*<0.05). Significant P values highlighted in bold indicate attraction while remaining significant P values indicate repulsion.

### Behavioral response of *D. citri* to synthetic chemicals

Behavioral bioassays with synthetic chemicals identified from Las-infected and non-infected plants revealed that *D. citri* were significantly attracted to MeSA at the 0.01 µg dosage, but that they were repelled by this chemical at the 100 µg dosage (Table 4). *D. citri* were attracted to D-limonene only at the 100 µg dosage, while MeAN did not attract or repel *D. citri* at any of the dosages tested. The positive control (β-ocimene) was attractive at 1–100 µg dosages (Table 4).

## Discussion

Our results indicate that Las-infected plants were initially more attractive to *D. citri* adults than non-infected plants; however, psyllids dispersed subsequently to non-infected plants after initially settling on infected plants. Similar results obtained with the behavioral responses of *D. citri* under both light and dark conditions suggest that initial movement of psyllids to Las-infected plants is likely mediated by volatile cues. Infection of citrus with this plant pathogen induced release of MeSA, which attracted *D. citri*. A similar amount of MeSA was also released by similar trees in response to *D. citri* infestation, suggesting that the same cue exploited by the pathogen to attract its vector may be used by the vector to locate congregations of conspecifics feeding on non-infected plants. Following initial chemically mediated attraction to infected plants, psyllids tended to subsequently disperse to non-infected plants, making them their preferred settling choice. We speculate that this may have been due to the sub-optimal quality of infected plants as compared with non-infected plants, as evidenced by lower feeding efficiency on infected than non-infected leaves. Importantly, this pathogen-manipulated behavioral sequence facilitated pathogen spread by the vector in a controlled laboratory experiment.

Our results suggest that an insect-transmitted bacterial plant pathogen alters plant traits so as to induce odor-mediated behavior from the vector that may ultimately benefit the pathogen. While increased preference of insect vectors to virus-infected versus non-infected host plants has been demonstrated for both persistently and non-persistently transmitted viruses [3], [4], [9], little is known about similar interactions involving pathogenic bacteria. Furthermore, most previous studies on pathogen-vector interactions have focused on single mechanisms (olfactory, visual or nutritional) [15]–[16], [61]–[64]. In the current investigation, we sought to determine several underlying mechanisms (chemical, visual, and nutritional) that affect the behavior of an insect (*D. citri*) in response to plant infection by the pathogen (*Candidatus* Liberibacter asiaticus) that it transmits.

Volatiles from infected and non-infected citrus are attractive to *D. citri* as confirmed by the current data and previous studies [40]. *D. citri* were attracted to common volatiles released by citrus such as β-ocimene and D-limonene, implicating these as general host selection cues. Both β-ocimene and D-limonene are predominant citrus volatiles released by citrus flush, foliage, and fruit [40], [65]–[67]. However, pathogen infection induced release of certain novel components or a quantitative increase in release of certain compounds that were released in lower quantities by non-infected plants. Among the novel chemicals identified that distinguished Las-infected from non-infected plants, only MeSA elicited attraction from *D. citri* and may explain the enhanced attractiveness of infected plants. However, the effect of MeSA on *D. citri* behavior, as part of a complex citrus volatile blend, requires further investigation. Specific blends of plant volatiles are important for attraction of herbivores to their host plants; however, individual components of such blends can act as repellents when not perceived within the context of the entire blend [68]–[69].

Movement of psyllids from infected to non-infected plants after initial selection of infected plants suggests that their initial response to olfactory cues may not be directly linked to the most beneficial host plant. This was also confirmed by feeding assays measured by honeydew excretion. These results suggest that psyllids must feed on infected plants in order to discern poor quality of the host. Therefore, volatiles appear to be involved in host finding, but not in arrestment on the host. Feeding is required for insects to differentiate between non-infected and infected plants and gustatory cues are involved in host acceptance [57], [70]–[71].

Insect herbivory can change the volatile profile of host plants. Insects that use a piercing/sucking mode of feeding have long-lasting interactions with plants cells and/or phloem [44]. It may not be surprising that plant responses to phloem-feeding are distinct from those occurring in response to chewing insects and that phloem-feeders often induce salicylate-dependent defense pathways commonly activated by bacterial, fungal, and viral pathogens [44], [56], [72]–[73]. A salicylate-dependent association is implicated in the current investigation given detection of MeSA release from plants infected with the Las pathogen. Production of MeSA is associated with the phytohormone, salicylic acid (SA), and its role in signaling plant defensive pathways, which regulate systemic acquired resistance against a wide variety of invading pathogens [74]–[75]. Zarate et al. [76] demonstrated that the whitefly, *Bemisia tabaci*, induces SA-signaling pathways, which suppress basal defenses constraining nymph development. Thus, attraction to plants with high levels of SA-signaling may benefit the herbivore. Furthermore, suppression of defenses via SA may be consistent with a number of strategies employed by phloem-feeding insects to avoid such defenses (see Walling [77]).

We investigated whether release of MeSA may be induced by *D. citri* feeding as a possible cue used by psyllids for locating established conspecifics or plants that have suppressed basal defenses. Volatile collections from psyllid-infested plants detected induced release of MeSA, suggesting a coincidental convergence on a single cue that may simultaneously benefit the pathogen by deceptively attracting its vector, which also uses this cue to locate conspecifics or identify vulnerable hosts. Broadly, these results are consistent with the “deceptive host phenotype” hypothesis proposed by Mauck et al. [9], given that release of an existing cue that attracts the vector (MeSA) to its host plant was also induced by pathogen infection, thus deceptively attracting the vector to nutritionally sub-optimal hosts.

Pathogen-induced release of plant volatiles has been previously established following virus infection. In these cases, CMV, PLRV, and BYDV induced release of volatiles that rendered infected plants more attractive to their aphid vectors than non-infected plants. Both PLRV and BYDV infections improved the quality of the host plants to their respective vectors [3], [4], [78]–[79], resulting in preferential arrestment on the infected plants due to contact and gustatory cues [3], [4], [58]. Our results are similar to the findings of Mauck et al [9], where squash plants infected with CMV released volatiles that initially attracted the aphid vector, but were poor hosts for their vectors, causing subsequent movement from infected to non-infected plants. Possible pathogen-mediated manipulation of vector behavior may depend on how pathogens are transmitted. Persistent pathogens, which require longer feeding durations for acquisition, benefit from increased arrestment of their vector, while non-persistent or semi-persistent pathogens may induce changes in plant phenotype that cause vectors to quickly disperse following acquisition [9]. In our experiments, psyllids were initially attracted to infected plants, but later preferred to settle on non-infected hosts. Therefore, our results with a bacterial plant pathogen appear to share similarities with previous results obtained with both non-persistent and persistent plant viruses. Although *D. citri* were initially attracted to infected plants, which was likely mediated by a volatile cue (consistent with both persistent and non-persistent virus examples [3], [9]), psyllids preferentially dispersed to non-infected plants after initial feeding on infected ones (consistent with non-persistent virus example [9]).

Pathogen spread may be favored in an environment with a low frequency of infected plants, when vectors prefer infected plants [80]. However, in an environment with a high frequency of infected plants, pathogen spread may be favored when vectors prefer non-infected plants [80]. More recent mathematical modeling similarly suggests that when vectors prefer to orient to infected plants, pathogen spread is rapid when the frequency of plant infection is low, but pathogen spread is slow when most plants are infected [57]. Moreover, vector preference for infected versus non-infected plants is partitioned into orientation preference and feeding preference and these two distinct suites of behavior affect pathogen spread differently, depending on whether vectors prefer infected versus non-infected plants [57]. While feeding preference for infected plants may decrease pathogen spread, orientation preference for infected plants may lead to rapid pathogen spread [57]. Acquisition of Las by *D. citri* can occur after 30 minutes to 24 hr of feeding [30], [33]. Therefore, initial landing by psyllids on infected plants for 24 hr or longer is sufficient for acquisition of the pathogen. Subsequent movement of psyllids from infected to non-infected plants will result in inoculation of new plants as demonstrated by the current results. Therefore, possible manipulation of *D. citri* behavior due to Las infection of plants may increase pathogen spread in the field; however, this will likely depend on the frequency of plant infection [57]. The effect of vector preference on pathogen spread depends on several other factors, including the mode of pathogen transmission, latency period, vector movement behavior, and the combined dynamic spatial patterns of the plant, pathogen, and vector [57], [80].

Las-infected plants were deficient in nitrogen, phosphorus, magnesium, zinc, and iron, but were characterized by higher concentrations of potassium and boron. It is possible that infected plants are a sub-optimal host for *D. citri* because of a nutritional imbalance caused by Las infection. Phloem-feeding plant hoppers (*Prokelisia dolus*) disperse to higher quality *Spartina* plants when reared on nitrogen and phosphorus deficient plants [81]. Also, increased nitrogen content improves the performance of leaf-feeding aphids (*Metopolophium dirhodum*) on wheat and barley [81]–[83]. Physiologically, nitrogen is essential for insect growth, survival, and reproduction due to its fundamental role in protein synthesis [84]–[88]. Phosphorus acts against viral disease by promoting plant maturity, thus restricting pathological effects of virus infection [89]. However, plant nutrient deficiencies can have a negative or positive effect on population dynamics of phloem feeding insects. For example, potassium deficiency in soybean increases fecundity and population growth of soybean aphid [90]–[92].

The nutrient analyses described in the current study only addressed the elemental composition of leaf tissues. There may be several other unidentified factors including sugars, amino acids, and proteins that could have contributed to the movement of *D. citri* from infected to non-infected plants. *D. citri* feed specifically within phloem cells, obtaining nutrition from free amino acids, proteins and sugars [28], which are known to affect vector growth [2], [71], [93]–[94]. Further comparisons of the elemental composition of psyllids and phloem tissues should help elucidate how host quality affects movement of *D. citri*. Las-infected leaves have been reported to accumulate up to 7.9-fold more starch than non-infected leaves, resulting in blockage of the phloem vessels [95]; however, the effect of starch accumulation on *D. citri* nutrition is still unknown. Starch accumulation and callose formation in rice leaves is reported to increase plant resistance to the brown plant hopper, *Nilaparvata lugens*, by preventing phloem ingestion [96]. In a similar experiment, plants infected with Zucchini yellow mosaic virus (ZYMV) were characterized by lower total protein and sugar content than non-infected plants [71]. However, both ZYMV-infected and non-infected plants had identical total amino acid contents and *Aphis gossypii* lived longer and produced more offspring on infected than on non-infected plants [71].

In summary, Las-infected plants were initially more attractive to *D. citri* adults than non-infected plants; however, psyllids dispersed subsequently to non-infected plants to make them their preferred location of settling rather than infected plants. Consistent with the “deceptive host phenotype” hypothesis [9], vectors were ‘deceptively’ attracted to the nutritionally suboptimal infected host plants during the initial settling period, which was of sufficient duration for *D. citri* to acquire the Las pathogen. Thus, the pathogen may be indirectly modifying the behavior of the vector by inducing changes in the attractiveness of the host plant through olfactory cues. This scenario suggests a mechanism for spread of the pathogen in the field because initial attraction and feeding of *D. citri* on infected host plants should facilitate acquisition of the pathogen, while subsequent movement away from potentially sub-optimal infected plants should facilitate inoculation of non-infected plants. Our results indicate that MeSA may be the specific chemical cue mediating initial psyllid attraction to Las-infected plants. It is possible that changes in nutritional content of plants stimulate subsequent dispersal of psyllids from infected to uninfected plants; however, this will require further investigation.

## Materials and Methods

### Maintenance of insect, pathogen and host plants

Non-infected adult *D. citri* used in behavioral bioassays were obtained from a laboratory culture at the University of Florida, Citrus Research and Education Center (Lake Alfred, USA). The culture was established in 2000 from field populations in Polk Co., FL, USA (28.0′N, 81.9′W) prior to the discovery of HLB in FL. The culture is maintained without exposure to insecticides on sour orange (*Citrus aurantium* L.) and ‘Hamlin’ orange [*C. sinensis* (L.) Osb.]. Monthly testing of randomly sampled *D. citri* nymphs, adults, and plants by quantitative real-time polymerase chain reaction (qPCR) assays is conducted to confirm that psyllids and plants in this culture are free of Las.

Las-infected *D. citri* were obtained from Las-infected *C. aurantium* and *C. sinensis* plants maintained in a secure quarantine facility at the University of Florida, Citrus Research and Education Center. Routine sampling indicated that about 70% of *D. citri* obtained from this colony were positive for Las when tested in qPCR assays. Both colonies were maintained at 27±1°C, 63±2% RH, and under a L14∶D10 hour photoperiod. Non-infected and Las-infected *D. citri* cultures were maintained in double-screened, 3.7×4.6 m secure enclosures located in separated buildings and with minimal risk of cross contamination.

Las infection in host plants was maintained by graft-inoculation of non-infected *C. sinensis* with Las-infected key lime (*Citrus aurantifolia*) budwood collected from citrus groves in Immokalee, FL, USA. Grafted plants were tested for Las infection by qPCR four months after grafting. Plants that tested positive for Las were used in experiments and for maintenance of Las cultures. Cultures of Las-infected plants were maintained through graft-inoculations because of low transmission efficiency of *D. citri* adults [19]. Because Las is not seed transmissible [97], Las-free host plants used in experiments were cultivated from *C. sinensis* seed or obtained as potted seedlings from an HLB-free commercial nursery to minimize the risk of undetectable latent infection of Las in grafted plants. The nursery-obtained plants were confirmed negative for Las infection by qPCR. All infected plants used for experiments exhibited minor or no symptoms, ranging from 0 to 1 on a graded symptom scale of 1 to 10. Non-infected and Las-infected plants were maintained in separate secure enclosures with minimal risk of cross contamination as described above.

### Detection of Las in insect and plant samples

Dual-labeled probes were used to detect Las in *D. citri* and citrus plants using an ABI 7500 system (Applied Biosystems, Foster City, CA) in a multiplex TaqMan qPCR assay described in [19], [98]. DNA from insect and plant samples was isolated using the DNeasy blood and tissue or DNeasy plant kits (Qiagen Inc, Valencia, CA), respectively. Las-specific 16S rDNA from psyllid and plant extracts was amplified using probe-primer sets targeting internal control sequences specific to *D. citri* [insect wingless] or plant [cytochrome oxidase] gene regions [19], [98]–[99].

DNA amplifications were conducted in 96-well MicroAmp reaction plates (Applied Biosystems). Quantitative PCR reactions consisted of an initial denaturation step of 95°C for 10 min followed by 40 cycles of 95°C for 15 s and 60°C for 60 s. Each 96-well plate containing *D. citri* samples included a no template control, a positive control (Las DNA in DNA extractions from *D. citri*), and a negative control (no Las DNA in DNA extractions from *D. citri*). Likewise, plates containing plant samples included a no template control, a positive control (Las DNA in DNA extractions from plant), and a negative control (no Las DNA in DNA extractions from plant). Reactions were considered positive for either target sequence if the cycle quantification (Cq) value, determined by the ABI 7500 Real-Time software (version 1.4, Applied Biosystems), was ≤32 [19].

### Response of psyllids to host plant odors

A custom designed two-port divided T-olfactometer [Analytical Research Systems (ARS), Inc. Gainesville, FL] that has been thoroughly described in [100] was used to evaluate the behavioral response of *D. citri*. Briefly, the olfactometer consisted of a 30 cm long glass tube with 3.5 cm internal diameter that is bifurcated into two equal halves with a Teflon strip forming a T-maze. Each half served as an arm of the olfactometer enabling the *D. citri* to make a choice between two potential odor fields. The olfactometer arms were connected to odor sources placed in 35 cm tall×15 cm wide dome shaped guillotine volatile collection chambers (GVCC) (ARS, Gainesville, FL) through Teflon-glass tube connectors. The odor sources comprised 14–16 week old Las-infected or non-infected *C. sinensis* plants placed into the GVCC. Purified and humidified air was pushed through the GVCC via two pumps connected to an air delivery system (ARS, Gainesville, FL). A constant airflow of 0.1 L/min was maintained through both arms of the olfactometer. The olfactometer was housed within a temperature-controlled room and positioned vertically under a fluorescent 900-lux light bulb within a fiberboard box for uniform light diffusion. This positioning took advantage of the negative geotactic and positive phototactic response of *D. citri* [100]. *D. citri* adults were released individually into the inlet adapter at the base of the olfactometer. An odor source was randomly assigned to one of the arms of the olfactometer at the beginning of each bioassay and was reversed after every 30 insects to eliminate positional bias. Initially, *D. citri* adults were exposed to clean air vs. clean air in the olfactometer to verify the absence of positional bias. Thereafter, the behavioral response of non-infected or Las-infected *D. citri* was tested against non-infected versus Las-infected plants. *D. citri* adults were also exposed to non-infected grafted (sham grafted) vs. non-grafted-non-infected plants to determine the effects of grafting only on *D. citri* behavior. For each treatment, *C. sinensis* plants were grafted with *C. aurantifolia* budwood four months prior to initiating behavioral experiments. A minimum of 150 male or female *D. citri* adults was examined per treatment combination (five replications of 30 *D. citri*). Each *D. citri* that responded to odors was individually subjected to qPCR to determine Las infection status using the procedures described above. Chi square (χ^2^) tests were used to compare between the numbers of *D. citri* entering the treatment or control arm in the T-maze olfactometer.

### Host plant selection under light or dark conditions

#### Light conditions

To evaluate settling preference of *D. citri* on non-infected versus Las-infected plants, two non-infected and two Las-infected plants of the similar age (14–16 week old) and vigor were placed randomly into a 0.35×0.35×0.6 m observation cage (Bioquip Products, Rancho Dominguez, USA). Thereafter, 240 *D. citri* adults (60 per plant) were released into the center of each cage. The cages were housed under temperature-controlled conditions of 27±1°C, 63±2% RH, and under a L14∶D10 h photoperiod. There were four cages with each cage representing a single replicate. The total number of *D. citri* settling on each Las-infected or non-infected citrus plant was recorded three and seven days after release. Experiments were conducted separately and in an identical manner for either non-infected or Las-infected *D. citri*. Each *D. citri* adult settling on a non-infected or Las-infected citrus plant was individually tested using qPCR to confirm Las infection status following the procedures described above. Citrus plants used in experiments were examined for Las infection four months after the completion of experiments.

#### Dark conditions

*D. citri* use a combination of olfactory and visual cues to locate citrus host plants [39]. Visual cues such as light and color of leaves may impact the settling preference of *D. citri* for non-infected versus Las-infected citrus plants. Therefore, an experiment was conducted under complete darkness to eliminate the role of visual cues. The materials and procedures were otherwise identical to those described above under light conditions. *D. citri* adults were counted three and seven days after release using a source of red light. Preliminary testing indicated that red light did not affect psyllid behavior. Furthermore, hemipterans lack red photoreceptors [101].

Numbers of *D. citri* settling on Las-infected versus non-infected citrus plants under light and dark conditions were analyzed using a repeated-measure, mixed-model, factorial analysis of variance (ANOVA) (Proc Mixed, Version 9.1, SAS Institute, Cary, NC, USA). Las infection status of citrus plants, Las infection status of *D. citri*, and gender of *D. citri* were included as fixed effects. Means were compared using Tukey's Honestly Significant Difference (HSD) test.

### Movement of *D. citri* between Las-infected and non-infected plants

Given that initial orientation of insect vectors to plants could be different from their final settling choices [57] and both could affect pathogen spread, we evaluated movement of *D. citri* between the Las-infected and non-infected plants. A non-infected or Las-infected plant was inserted at random into an above described observation cage. Thereafter, 60 *D. citri* adults were confined on this plant using a mesh sleeve for forced settling. Once all psyllids settled on the plant, an additional, randomly selected non-infected or Las-infected plant was inserted into the cage and placed 15 cm away from the initial plant. Immediately thereafter, the mesh sleeve was removed from the initial plant to allow psyllid movement between the two plants. The number of *D. citri* moving from the initial plant on which they settled and onto the inserted plant was counted seven days after release. Four combinations of plant pairs were examined using either non-infected or Las-infected *D. citri* for a total of eight treatments. The plant combinations tested were: 1) Las-infected settling plant with a non-infected plant introduction, 2) non-infected settling plant with a Las-infected plant introduction, 3) non-infected settling plant with a non-infected plant introduction, and 4) Las-infected settling plant with a Las-infected plant introduction. The cages were housed in a temperature-controlled room at 27±1°C, 63±2% RH, and under a L14∶D10 h photoperiod. Each combination was replicated four times. Non-infected *D. citri* adults found moving from infected to non-infected plants were individually analyzed for Las infection by qPCR as described above. Thereafter, the inserted plants were subsequently tested for nutritional status and possible Las infection due to movement of infected psyllids. Only female psyllids were used in these experiments, because no differences were observed in behavioral responses between the sexes in preliminary tests. A two-way ANOVA was used to analyze the number of *D. citri* adults moving between plants, with psyllid status (infected or non-infected) and plant treatment pair serving as factors. Means were subsequently separated using Tukey's HSD test. *D. citri* adults that did not make a choice were excluded from statistical analyses.

### Nutritional status of Las-infected and non-infected citrus plants

The nutritional status of Las-infected and non-infected citrus plants was analyzed by a commercial laboratory (Waters Agricultural Lab, Inc, Georgia). Leaf samples from 16 qPCR-confirmed Las-infected or non-infected plants were washed, dried and ground to pass a 0.38-mm sieve. An individual leaf sample comprised about three g of dry leaf biomass obtained from three-month-old leaves. Leaf phosphorus, potassium, calcium, magnesium, sulfur, manganese, iron, copper, zinc, boron, silicon, sodium, molybdenum, nitrate, aluminum, and chlorine concentrations were determined by inductively coupled plasma atomic emission spectroscopy. The nutrient data obtained from Las-infected versus non-infected plants were analyzed using two-sample t-tests.

### Feeding efficiency of *D. citri* on non-infected versus infected plants

The objective of these experiments was to compare feeding efficiency of *D. citri* adults on Las-infected versus non-infected plants by quantifying their honeydew excretion. Four sets of experiments were conducted to quantify honeydew excretion of psyllids after 24, 48, 72 or 96 hr of feeding. Each experiment was replicated five times. Citrus leaf discs obtained from Las-infected or non-infected plants were placed individually on 1.5% agar beds within 60 mm plastic disposable Petri dishes. Thereafter, ten *D. citri* adults of mixed age and sex (∼1∶1) were released into each dish. Each Petri dish was sealed with lids lined with 60 mm Whatman filter paper (Whatman International Ltd, Kent, UK). Petri dishes were inverted to collect honeydew droplets onto the filter paper. Dishes were maintained at 25±1°C, 50±5% RH, and L14∶D10 h photoperiod in an incubator. Collected filter papers were subjected to a ninhydrin (Sigma-Aldrich, St Louis, MO) test to facilitate counting of honeydew droplets [102]–[103]. Significant differences in the number of honeydew droplets excreted on Las-infected versus non-infected leaves were determined by a two-way ANOVA with Las infection status of leaves and *D. citri* exposure period as independent factors. Means were compared using Tukey's HSD tests. This technique, developed by Auclair [104], has been successfully used to quantify honeydew excretion by psyllids, whiteflies, aphids and plant hoppers [102]–[103], [105]–[106].

### Collection of plant volatiles from Las-infected or non-infected citrus plants

Infected or non-infected plants, as described above, were confined within a GVCC (ARS, Gainesville, FL). Charcoal-purified and humidified air was drawn over plants and pulled out at a rate of 300 mL min-1 through a trap containing 50 mg of Super-Q adsorbent, 800–1000 mesh (Alltech Assoc., Deerfield, Illinois, USA) for 24 hrs. Thereafter, Super-Q traps were rinsed with 150 µl of dichloromethane into individual 2.0 ml clear glass vials (Varian, Palo Alto, CA, USA, part number: 392611549 equipped with 500 µl glass inserts). Volatiles were sampled from two plants simultaneously (one Las-infected and the second non-infected). A total of five plants that were previously identified as infected and five similar plants identified as non-infected were sampled.

### Analysis of volatiles by GC-MS

An internal standard of n-octane (300 ng) was added to each sample prior to GC-MS analysis. A one µl aliquot of each extract was injected onto a gas chromatograph (HP 6890) equipped with 30 m×0.25-mm-ID, 0.25 µm film thickness DB-5 capillary column (Quadrex, New Haven, CT, USA), interfaced to a 5973 Mass Selective Detector (Agilent, Palo Alto, CA, USA), in both electron impact (EI) and chemical ionization (CI) modes. Helium was used as the carrier gas in the constant flow mode of 30 cm/sec. The injector was maintained at 260°C. The oven was programmed from 40 to 260°C at 7°C/min. Isobutane was used as the reagent gas for CI, and the ion source temperature was set at 250°C in CI and 220°C in EI. The mass spectra were matched with NIST 2005 version 2.0 standard spectra (NIST, Gaithersburg, MD). The compounds with spectral fit values equal to or greater than 90 and appropriate LRI values were considered positive identifications. When available, mass spectra and retention times were compared to those of authentic standards. Compounds were quantified as equivalents of the total amount of n-octane within each analyzed volatile collection sample. A more accurate quantification of MeSA was made by comparing the total ion chromatograph (TIC) EI response for standard solutions with known quantities of n-octane and MeSA. A response factor of 1.2 was then established for MeSA relative to the n-octane based values.

The resulting volatile profiles were standardized as equivalents of n-octane within each sample analyzed. The characteristic set of variables that defined a particular group (e.g. non-infected versus infected plant) was found using the ‘varSelRFBoot’ function of the package ‘varSelRF’ for the ‘randomForest’ analysis (R software version 2.9.0, R Development Core Team 2009). We used the *varSelRF* algorithm with Random Forests to select the minimum set of VOCs that were characteristic of differences between infected and non-infected plants. The tree-based Random Forests algorithm performs hierarchical clustering via multi-scale and combinatorial bootstrap resampling and is most appropriate for data where the variables (VOCs in this case) outnumber the samples, and where the variables are auto correlated, which is a typical problem of conventional multivariate analysis of such data. Consequently, this type of analysis is common in bioinformatics, chemoinformatics and similar data-rich fields. We employed 200 bootstrapping iterations of the Random Forests algorithm to arrive at a minimal set of VOCs that could differentiate between infected and non-infected plants. We also calculated the mean decrease in accuracy (MDA) when individual VOCs are removed from the analysis. MDA values indicate the importance value of particular VOCs for the discrimination between treatments.

### Collection of volatiles from citrus plants before and during psyllid feeding

Volatiles were collected to determine whether release of MeSA was induced by psyllid feeding. Plants were placed within a GVCC with identical flow rates and collection procedures as described above. Volatiles were collected simultaneously from three undamaged plants (no psyllid feeding) for 24 hrs, after which the adsorbent traps were rinsed into vials for gas chromatography–mass spectrometry (GC-MS) analysis as described above for the collection of volatiles from infected and uninfected plants. Psyllids were divided by sex and randomly introduced into two of the three plant chambers. Each infested plant received 300 psyllids (one plant receiving only males and another plant receiving only females). The third plant was left uninfested as a control. Volatiles were collected from plants for 24 hr, after which traps were rinsed into vials for GC-MS analysis. Volatiles were collected in this manner on three separate dates with six plants evaluated before and after *D. citri* introduction and three plants serving as simultaneous undamaged controls. VOCs that were characteristic of differences between plants before and after psyllid feeding were determined as described above. Treatment differences between individual volatiles collected from headspace of infected and non-infected plants were compared using two-sample t-tests.

### Behavioral response of *D. citri* to synthetic chemicals

MeSA was released in greater quantities from Las-infected plants than non-infected plants, while MeAN and D-limonene were released in greater quantities by non-infected than infected plants (See results). Therefore, the behavioral response of *D. citri* to these synthetic chemicals was tested. A known *D. citri* attractant, β-ocimene [40], was used as a positive control. All chemicals were obtained from Sigma Aldrich (St Louis, MO) with purities ranging between 97 and 99%. Purity of β-ocimene was ≥90% and contained a mixture of isomers comprising 20–25% limonene. Each treatment was dissolved in 100 µl of dichloromethane and pipetted onto a 2 cm Richmond cotton wick (Petty John Packaging, Inc. Concord, NC) at 0.001, 0.01, 0.1, 1.0, 10.0 and 100.0 µg dosages. The control treatment consisted of a cotton wick impregnated with solvent only. The solvent from both treatments was allowed to evaporate within a fume hood for 30 min prior to assays. The bioassay procedures with synthetic chemicals were identical to those described earlier for plant samples. However, in this case, treatments (odor sources) were placed into solid phase micro extraction chambers (SPMEC) (ARS, Gainesville, FL) instead of the GVCC described above. The SPMEC consists of a straight glass tube (17.5 cm long×2.5 cm internal diameter) supported with an inlet and outlet valve for incoming and outgoing air streams, respectively [102]. The treated and control cotton wicks were wrapped in laboratory tissue (Kim wipes, Kimberly-Clark, Roswell, GA) to minimize contamination and placed randomly into one of the two SPMEC enclosures that were connected to the olfactometer and the air delivery system through Teflon-glass tube connectors. Only non-infected female psyllids were assayed in these experiments, because no differences in behavioral responses were observed between the sexes or between infected and non-infected psyllids in preliminary tests. At least 120 non-infected female adults were examined per treatment combination. The treatment combinations evaluated in this set of experiment were 1) MeAN vs. clean air, 2) MeSA vs. clean air, 3) D-limonene vs. clean air, and 4) β-ocimene vs. clean air. Chi square (χ^2^) tests were used to compare between the numbers of *D. citri* entering the treatment or control arm in the T-maze olfactometer.
